# Novel Graphene Oxide/Quercetin and Graphene Oxide/Juglone Nanostructured Platforms as Effective Drug Delivery Systems with Biomedical Applications

**DOI:** 10.3390/nano12111943

**Published:** 2022-06-06

**Authors:** Alexa-Maria Croitoru, Alina Moroșan, Bianca Tihăuan, Ovidiu Oprea, Ludmila Motelică, Roxana Trușcă, Adrian Ionuț Nicoară, Roxana-Cristina Popescu, Diana Savu, Dan Eduard Mihăiescu, Anton Ficai

**Affiliations:** 1 Department of Science and Engineering of Oxide Materials and Nanomaterials, Faculty of Applied Chemistry and Materials Science, University Politehnica of Bucharest, Gh. Polizu St. 1–7, 011061 Bucharest, Romania; alexa_maria.croitoru@upb.ro (A.-M.C.); bianca.tihauan@sanimed.ro (B.T.); ovidiu.oprea@upb.ro (O.O.); ludmila.motelica@upb.ro (L.M.); roxana_doina.trusca@upb.ro (R.T.); adrian.nicoara@upb.ro (A.I.N.); roxana.popescu@nipne.ro (R.-C.P.); anton.ficai@upb.ro (A.F.); 2 National Centre for Micro- and Nanomaterials, University Politehnica of Bucharest, Spl. Independentei 313, 060042 Bucharest, Romania; 3 National Centre for Food Safety, University Politehnica of Bucharest, Spl. Independentei 313, 060042 Bucharest, Romania; 4 Department of Organic Chemistry Costin Nenitescu, Faculty of Applied Chemistry and Materials Science, University Politehnica of Bucharest, Gh. Polizu St. 1–7, 011061 Bucharest, Romania; alina.morosan@upb.ro; 5 Research Institute of the University of Bucharest—ICUB, Spl. Independentei 91-95, 0500957 Bucharest, Romania; 6 Research & Development for Advanced Biotechnologies and Medical Devices, SC Sanimed International Impex SRL, 087040 Călugăreni, Romania; 7 Academy of Romanian Scientists, Ilfov St. 3, 050045 Bucharest, Romania; 8 “Horia Hulubei” National Institute for Research & Development in Physics and Nuclear Engineering, Reactorului, St. 30, 077125 Magurele, Romania; dsavu@nipne.ro

**Keywords:** graphene oxide, drug delivery systems, quercetin, juglone, cancer therapy, antimicrobial activity

## Abstract

In this paper, novel drug delivery systems (DDS) were designed based on graphene oxide (GO) as nanocarrier, loaded with two natural substances (quercetin (Qu) and juglone (Ju)) at different concentrations. The chemical structure and morphology of the synthesized GO-based materials were characterized by Fourier transform infrared spectroscopy (FTIR), scanning electron microscopy (SEM), and Raman spectroscopy. The antibacterial activity was evaluated against standard strains, *Staphylococcus aureus* ATCC 6538, *Escherichia coli* ATCC 8739, and *Candida albicans* ATCC 10231. Results demonstrated excellent antimicrobial activity, with a 5 log reduction of *E. coli* and a 1 log to 3.04 log reduction of *S. aureus* populations. Reduction rates were above 90%. Biocompatibility tests were also performed on GO-based materials, and the results showed biocompatible behavior for both L929 fibroblast cell line and BT474 breast cancer cells at lower concentrations. The identity of Qu and Ju was demonstrated by matrix-assisted laser desorption/ionization (MALDI) analysis, showing the compounds’ mass with high accuracy. In addition, specific properties of GO made it a versatile matrix for the MALDI analysis. The results of this study indicated that GO-based platforms may be suitable for applications in many areas for the effective and beneficial use of hydrophobic compounds such as Ju and Qu.

## 1. Introduction

Nanomedicine has recently focused on developing innovative DDSs for the transport of natural substances, with minimal adverse effects on the human body, in order to overcome the limitation in the bioavailability of these natural products and to improve the efficacy of these natural agents to allow the introduction of new therapies [1]. The next generation of DDSs based on nanostructured materials and nanocomposites have been proven to have extraordinary benefits in terms of solubility, bioavailability, toxicity, pharmacological activity, stability, targeted delivery, and both physical and chemical degradation [2,3,4,5].

Different functional nanocarriers (such as carbon-based materials, metal and metal oxide nanoparticles, polymeric nanoparticles, metalloids, etc.) have been obtained and successfully applied in the delivery of various biological agents [6]. GO has gained much attention in biomedical applications due to its biocompatibility and promising properties, such as high specific surface area, ability to realize hydrogen bonds, π-π stacking, electrostatic and hydrophobic interactions with the biological active agents, mechanical strength, thermal stability, and electrical and optical properties [7,8,9,10]. In the past few years, it was found that GO has an excellent ability to immobilize many substances, including metals, drugs, biomolecules, and fluorescent molecules [2,9,11] with anticancer, antibacterial, and/or antiviral properties [3,12].

Quercetin is a natural flavonoid that has been found to show several pharmacological activities, such as antitumor, antioxidation, antibacterial, and anti-inflammation [13,14,15]. Juglone (5-hydroxy-1,4- naphthoquinone) is an allelopathic compound that is mostly found in the roots, leaves, nut-hulls, bark, and wood of walnut (*Juglans regia*). It has been studied for its antitumor and antimicrobial activities [16,17]. Sarkar et al. [18] synthesized folic acid (FA)-armed mesoporous silica nanoparticles (MSN-FA-Qu) loaded with Qu and demonstrated that Qu inhibited breast cancer cells and induced cell cycle arrest. An efficient DDS was designed using modified GO with biocompatible hyperbranched polyglycerol (HPG), non-covalently loaded with Qu. The results demonstrated up to 185% drug-loading capacity and up to 93% encapsulation efficiency. The release profile of Qu was controlled and sustained under various pH levels. In addition, HPG-GO did not show any cytotoxicity on the MCF7 cell line in different concentrations for 72 h of incubation [19].

The development of novel biotechnological approaches in the biomedical field seems to be at an all-time high. However, as new solutions appear, further challenges arise. The bacterial colonization of medical devices continues to be a key issue for the health care system [20]. Every current therapeutic certainly approach takes this into consideration, but as we have learned by now, microorganisms have the capacity of evolving as fast as our technological creativeness does. Therefore, novel solutions and compounds need to be utilized in order to keep up with the evolution of bacterial resistance mechanisms. A prevalence of *S. aureus, E. coli,* and *C. albicans* infections associated with the biomedical field has been reported by several authors [20,21,22,23,24,25,26,27,28,29].

The present paper investigated the release profile, antimicrobial activity, and biocompatibility of nanostructured platforms containing GO as nanocarrier loaded with Qu and Ju as biological active substances with anticancer and antimicrobial activities. Moreover, this study focusses on the role of GO-based materials in various disease therapies, showing the promising future of nanomedicine.

## 2. Materials and Methods

Graphene oxide powder (~4.2 nm in thickness, ~10–20 layers) was obtained by Hummers modified method [30]. Quercetin ≥95% (HPLC) solid (Sigma Aldrich, Taufkirchen, Germany), juglone 97% (Sigma Aldrich, Taufkirchen, Germany), sodium chloride, sodium bicarbonate, magnesium chloride hexahydrate, hydrochloric acid 36.5–38%, sodium sulfate (Silal Tradding, Bucharest, Romania), potassium chloride, dibasic potassium phosphate trihydrate, calcium chloride (Sigma Aldrich, Taufkirchen, Germany), and tris-hydroxymethyl aminomethane (Serva Electrophoresis GmbH, Heidelberg, Germany) were used in this paper.

### 2.1. Fabrication of GO-Based Materials

GO was obtained using modified Hummers method, presented in our previous paper [30]. In order to obtain GO loaded with Qu (GOQu) and Ju (GOJu), different Qu and Ju solutions were prepared using ethanol as solvent. Into each solution, 0.2 g GO was added, and the mixtures were stirred at room temperature until the solvent evaporated. The nanomaterials were dried in a vacuum oven at 40 °C for 24 h. The final concentrations of the nanocomposite were: GOQu 5% (*w*/*v*), GOQu 2.5% (*w*/*v*), GOJu 10% (*w*/*v*), and GOJu 5% (*w*/*v*).

### 2.2. Sample Preparation by Dip-Coating Process for MALDI Analysis

The GOQu and GOJu nanofilms were obtained by dip-coating. Different suspensions were prepared in a Berzelius glass, by dissolving known amounts of Qu and Ju in ethanol and adding 20 mg of GO. The final concentrations of the nanocomposite were: GOQu 5% (*w*/*v*), GOQu 2.5% (*w*/*v*), GOJu 10% (*w*/*v*), and GOJu 5% (*w*/*v*). Then, indium-tin-oxide (ITO)-coated glass slides (Bruker Daltonics, Bremen, Germany) were dipped into matrix-containing suspensions. A thin uniform film was applied to the surface of the ITO-coated glass slides by solvent evaporation.

The matrix-assisted laser desorption/ionization high-resolution mass spectrometry (MALDI-HRMS) analyses were performed by a Bruker SolariX-XR 15T Fourier transform ion cyclotron resonance (FT-ICR) system, using 100 µm steps for the surface scan raster.

### 2.3. In Vitro Drug Release Studies

To study the release profile of the loaded agents onto GO nanocarrier, a simulated body fluid solution (SBF) with a pH of 7.4 was used. The SBF was prepared in accordance with the original protocol published by Kokubo et al. [31]. The method used for determining the release profile of the drugs was sample and separate (SS). About 80 mg of each nanomaterial was added into 50 mL of SBF. From time to time (0.5, 1, 2, 5, 8, 24, 48, 72, 144, and 240 h respectively), 2 mL of sample solution was taken, replaced with an equal amount of the fresh medium, and analyzed by UV–Vis in order to measure the released profile of the substances. During this time, the solutions were kept in the oven at 37 °C.

A UV–Vis JASCO (Easton, PA, USA) V560 spectrophotometer was used to measure the amount of active substances released into the SBF. Absorbance values were measured in standard quartz cuvettes at 290 nm for GOJu and 253 nm for GOQu, respectively, and quantification was based on calibration curves realized for the two polyphenols.

### 2.4. Characterization of the Scaffolds

The synthesized GO-based materials were characterized by Fourier transform infrared spectroscopy (FTIR) using a Nicolet iS50FT-IR (Thermo Fisher Scientific, Waltham, MA, USA) spectrometer equipped with a DTGS detector, which provides information with a high sensitivity in the range of 4000 cm^−1^ and 500 cm^−1^ at a resolution of 4 cm^−1^. All spectra were registered in attenuated total reflectance (ATR) mode using a diamond crystal.

The surface morphological characterizations of the GO-based materials were determined by scanning electron microscopy (SEM). The images were obtained using a Quanta Inspect F50 electron microscope (Eindhoven, The Netherlands) equipped with a field emission electron source (FEI Inspect F50, Eindhoven, The Netherlands), with a resolution of 1.2 nm at 30 kV and 3 nm at 1 kV (BSE).

Raman spectroscopy analyses were performed using Horiba equipment (Labram HR Evolution, Pailaiseau, France), with an excitation wavelength of 514 nm and a 50× objective with a 10 s acquisition time.

### 2.5. Assessment of Antimicrobial Activity

Semi-quantitative and quantitative methods were used for assessment of antimicrobial activity. The logarithmic and percentage reduction, recovery rate, and antimicrobial susceptibility were assessed. When developing and evaluating new materials, recovery rate (%) represents a very effective screening method. When analyzing the antimicrobial activity of new materials (with potential use as medical devices), this demonstration is critical in the accurate determination of antimicrobial, disinfecting efficacy, bioburden, sterility, or in any test that requires determination of surviving microorganisms in a product containing antimicrobial properties. Failure to confirm adequate neutralization and recovery could result in under-reporting of surviving microorganisms. Tested samples were GOQu 2.5% (*w*/*v*), GOQu 5% (*w*/*v*) (quercetin loaded on graphene oxide), GOJu 5% (*w*/*v*), and GOJu 10% (*w*/*v*) (juglone loaded on graphene oxide).

All tests were performed using three reference strains from the American Type Culture Collection (ATCC, Manassas, VA, USA): *Staphylococcus aureus* ATCC 6538, *Escherichia coli* ATCC 8739, and *Candida albicans* ATCC 10231. Microbial susceptibility was assessed according to CLSI 2018 M07 [32]. The microbial suspensions of 1.5 × 10^8^ CFU/mL, obtained from fresh 15 to 18 h cultures and developed on solid medium, were adjusted using the nephelometric McFarland standard at 0.5 for bacteria and 1 for fungi, and then serially diluted to 10^−5^. The inoculum volume was adjusted according to the samples’ mass. The samples were placed in contact with the microbial inoculum for 30 min and thoroughly spun on a vortex, and afterwards, 5 decimal serial dilutions were carried out in order to determine the logarithmic and percentage reduction of the microbial populations. Then, 10 µL in triplicate was inoculated in a spot on Muller Hinton solid medium, or Sabouraud for microfungi. After 18–24 h of incubation at 36 ± 2 °C, the plates were read by counting the colonies.

The logarithmic reduction was calculated using the formula:

Logarithmic reduction = Logarithmic reduction = lg A/B(1)

where A is the number of viable organisms before treatment, and B is the number of viable organisms after treatment.

The percentage reduction in microbial populations was calculated using the formula:

P = (1−(B/A)) ×100(2)

where P is the percentage reduction, and L the logarithmic reduction.

The determination of the recovery rate was carried out by performing 12 decimal serial dilutions from the inoculated samples with 10^−5^, after 18 to 24 h of incubation. The recovery factor was calculated using the formula:

(3) RF=CFU positive control/CFU sample

The number of colonies obtained for the sample was compared with those obtained on the control plates. The numbers of colonies counted should not differ by more than a factor of 2 (recovery rate 50–200%).

Statistical analysis was performed using GraphPad Prism 9 (San Diego, CA, USA). Data were analyzed using the two-way ANOVA test. The level of significance was set to *p* < 0.05.

### 2.6. Assessment of Antimicrobial Susceptibility by Broth Dilution Method

The antimicrobial susceptibility of the sample suspensions was assayed on Gram-negative (*Escherichia coli* ATCC 8739), Gram-positive (*Staphylococcus aureus* ATCC 6538), and microfungi (*Candida albicans* ATCC 10231) reference strains obtained from the American Type Culture Collection (ATCC, Manassas, VA, USA). Microbial suspensions of 1.5 × 10^8^ CFU/mL (0.5 McFarland density) obtained from 15 to 18 h bacterial cultures developed on solid media were used. The samples were suspended in distilled water in order to prepare a stock solution of 200 mg/mL concentration. The quantitative assay of the antimicrobial activity was performed by the microdilution method in 96 multi-well plates. The following concentrations were tested: 200 mg/mL, 100 mg/mL, 25 mg/mL, 3.125 mg/mL, and 0.195 mg/mL. Culture positive controls (wells containing culture medium seeded with the microbial inoculum) were used. The plates were incubated for 24 h at 37 °C, the absorbance was assessed at 620 nm using a microplate spectrophotometer, and the effective antimicrobial concentrations (lowest cytotoxicity, highest antimicrobial effect) were determined by comparison with positive control and GO control results. Sample analysis was performed in triplicate. Statistical analysis was performed using GraphPad Prism 9 (San Diego, CA, USA). Data were analyzed using the two-way ANOVA test. The level of significance was set to *p* < 0.05. The EC50 (half maximal effective concentration) was calculated using AAT Bioquest online (*AAT Bioquest, Inc. Quest Graph™ LD50 Calculator,* Sunnyvale, CA, USA). The therapeutic index was calculated for each sample as the ratio between the EC50 in case of antimicrobial susceptibility values for each strain and the EC50 in case of biocompatibility with normal cells (L929).

### 2.7. Biocompatibility Tests

In order to determine the biological effect of the GO nanoparticles, the L929 fibroblast cell line (ATCC) and BT474 breast cancer cell line (CLS) were used. The L929 cells were cultured in Earle’s minimum essential medium (MEM) containing L-glutamine (Biochrom, Merck Millipore, Darmstadt, Germany) supplemented with 10% fetal bovine serum (FBS) and 1% penicillin and streptomycin antibiotics, in standard conditions of temperature and humidity (37 ± 2 °C, 5 ± 1% CO_2_, and more than 90% humidity). The BT474 cells were cultured in Dulbecco’s Modified Earle’s medium (DMEM) containing L-glutamine (Biochrom, Merck Millipore, Darmstadt, Germany) supplemented with 10% fetal bovine serum (FBS) and 1% penicillin and streptomycin antibiotics, in standard conditions of temperature and humidity (37 ± 2 °C, 5 ± 1% CO_2_, and more than 90% humidity). The samples were suspended in ultrapure water at a concentration of 10 mg/mL and sterilized using γ-irradiation using a ^60^Co source (SVST Co-60/B irradiator, Institute of Isotopes Co. Ltd. Budapest, Hungary), at a dose higher than 25 kGy.

The cellular viability and proliferation were quantitatively measured using the MTT tetrazolium-salt assay (Serva Electrophoresis GmbH, Heidelberg, Germany). Thus, 5000 cells of each type were seeded in each corresponding well and incubated in standard conditions over 4 h to allow their attachment. After this time, the culture medium was replaced with GO-containing medium at different concentrations (binary dilutions of 250 μg/mL) and incubated for an additional 48 h. At the corresponding time-point, the medium was removed and gently replaced with fresh culture medium containing 10% MTT solution (5 mg/mL in PBS). The cells were incubated for another 3 h in standard conditions, and afterwards, the supernatant was replaced with DMSO, in order to solubilize the grown formazan crystals. The absorbance corresponding to each sample was measured at 570 nm wavelength using a microplate reader.

All experiments were performed in triplicate, and the data was presented as mean ± SD and represented as dependent on the drug concentration decimal logarithm. The dose–response fit was obtained using the specific function implemented in OriginPro 8.6 software (OriginLab Corporation, Northampton, MA, USA). The statistical analysis was performed using a two-tailed Student’s test, where values of *p* < 0.05 were considered as statistically significant. The EC50 (half maximal effective concentration) was calculated using the AAT Bioquest online LD50 calculator (*AAT Bioquest, Inc. (2021, May 28). Quest Graph™ LD50 Calculator, Sunnyvale, CA, USA.”* Retrieved from https://www.aatbio.com/tools/ld50-calculator, access date: 15 January 2022). The therapeutic index was calculated for each sample as the ratio between the EC50 in case of tumor cells (BT474) and the EC50 in case of normal cells (L929).

## 3. Results and Discussions

This report presents the characterization of the GO-based materials designed by loading GO with natural substances (Qu and Ju).

### 3.1. Fourier Transform Infrared Spectroscopy (FTIR)

**Figure 1:**
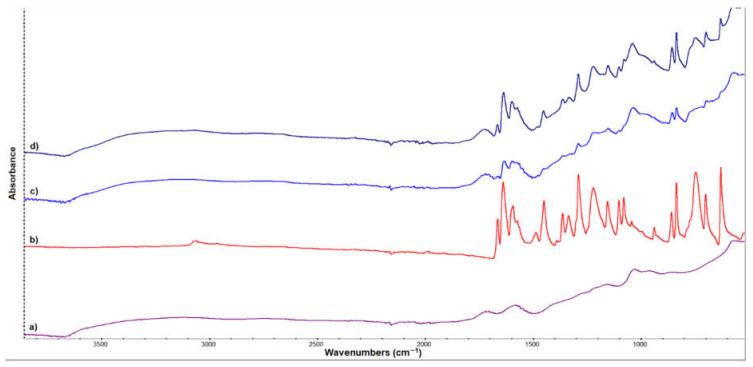
FTIR spectra for (**a**) GO, (**b**) Ju, (**c**) GOJu 5% (*w*/*v*) (juglone loaded onto GO nanocarrier), and (**d**) GOJu 10% (*w*/*v*).

FTIR spectra of GO (shown in Figure 1a) indicate the main characteristic peaks of GO, which are similar to those found in literature reports [33,34,35]. The signal from 1711 cm^−1^ is attributed to C = O stretching vibration presented in the carbonyl and carboxyl groups of GO. The peak around 1600 cm^−1^ is assigned to C = C stretching vibration, and the band between 1240 cm^−1^ and 1000 cm^−1^ corresponds to COC (epoxy) and alcohol groups. The broad absorption band centered at 3123 cm^−1^ is attributed to the associated OH group from GO.

Figure 1b presents the characteristic peaks of Ju. The signals between 3000 and 3100 cm^−1^ refer to the stretching vibration of the -CH bonds. The peaks from 1662 and 1636 cm^−1^ are attributed to the non-hydrogen bonded carbonyl stretching frequency and intermolecular hydrogen bonded carbonyl stretching vibration, respectively. The stretching vibration of aromatic skeletal structure occurs between 1470 and 1610 cm^−1^. The two peaks at 1287 cm^−1^ and 1228 cm^−1^ represent the C-OH stretching and bending vibrations. The signal from 698 cm^−1^ could be attributed to the -CH out-of-plane bending vibration of the alkenes, and the signals from 460 and 627 cm^−1^ show the C-C benzene ring out-of-plane bending vibration [36,37].

After the loading of GO with Ju, FTIR spectra of GOJu showed the characteristic signals of the drug, but these are broadening because of the interaction with the GO support. A difference in relative intensity can be also observed, demonstrating the successful formation of the complex. Comparing GOJu 5% (*w*/*v*) with GOJu 10% (*w*/*v*), an increase in the relative intensity of the peaks can be observed due to the higher Ju concentration [36]. It is important to mention that even if 10% (*w*/*v*) of Ju is present, the peaks of Ju are not clearly identified, which means that strong interactions between GO and Ju appear.

**Figure 2:**
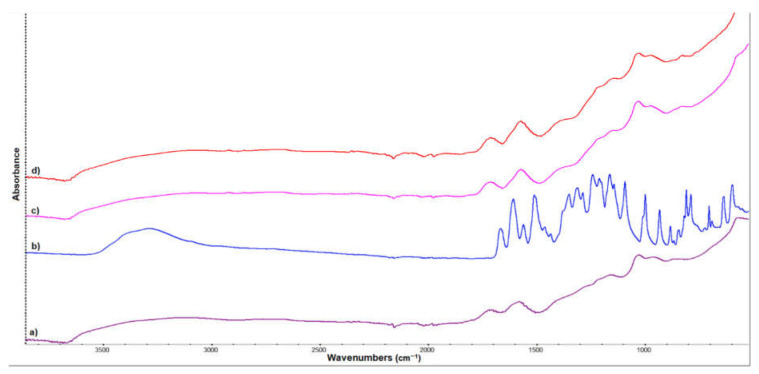
FTIR spectra for (**a**) GO, (**b**) Qu, (**c**) GOQu 2.5% (*w*/*v*) (quercetin loaded onto GO nanocarrier), and (**d**) GOQu 5% (*w*/*v*).

The FTIR spectrum of pure Qu is showed in Figure 2b. The broad band at 3287 cm^−1^ is assigned to the -OH stretching vibration. The signal from 1664 cm^−1^ refers to C = O carbonyl functional groups, and the peaks detected at 1605, 1559, and 1509 cm^−1^ belong to the C = C aromatic bonds stretching. The C-H stretching vibration in aromatic hydrocarbon occurs at 1311 cm^−1^. The signals between 1270 and 1028 cm^−1^ correspond to CO stretching in the aryl ether ring [38,39,40,41]. The FTIR spectrum of the complex GOQu showed no significant changes. An increase in the relative intensity of the peaks around 1600 cm^−1^ and 1020 cm^−1^ can be observed due to the increase in the Qu concentration [42], providing evidence of controlled release.

### 3.2. Scanning Electron Microscopy (SEM)

**Figure 3:**
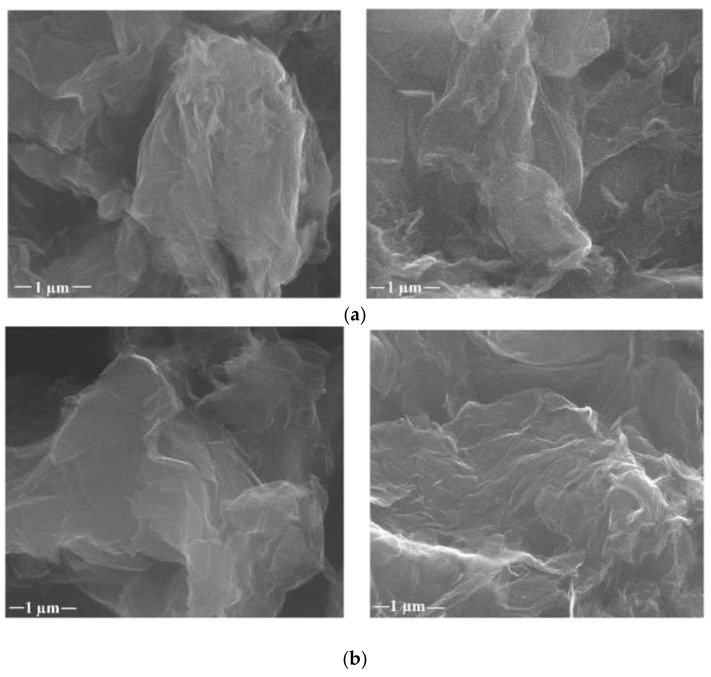
SEM images for (**a**) GOJu 10% (*w*/*v*) (**left**), GOJu 5% (*w*/*v*) (**right**), (**b**) GOQu 5% (*w*/*v*) (**left**), and GOQu 2.5% (*w*/*v*) (**right**) at 100,000× mag.

The surface morphology of GO loaded with biological active agents was characterized by SEM analysis, as shown in Figure 3. Higher-magnification images were presented for a better visualization of the graphene. The morphology of GO sheets showed a very thin and lamellar structure on the macrolevel, but the folded shape can also be noticed because of the high degree of functionalization [30,43,44]. When GO was loaded with Qu and Ju, respectively, no significant changes were observed, GOQu and GOJu having an apparently smooth and irregular surface. Nevertheless, a slight increase in the degree of particle agglomeration can be observed in Figure 3a,b because of the presence of the active compounds on the GO support, giving them a slightly irregular form [45,46,47]. No agglomerates can be seen on the surface, which means that both polyphenols are adsorbed well and continuously onto the GO. This fact provides evidence of controlled release.

### 3.3. Raman Spectroscopy

**Figure 4:**
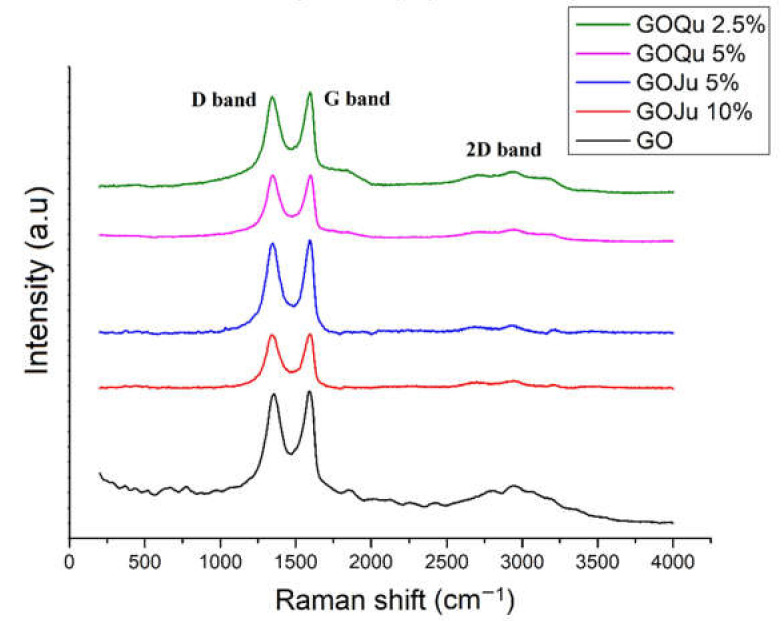
Raman spectra of GOQu 5% (*w*/*v*), GOQu 2.5% (*w*/*v*), GOJu 10% (*w*/*v*), GOJu 5% (*w*/*v*), and GO material.

Raman spectroscopy was used to examine the defects and disorder in the structure of GO and GO-based drug delivery systems loaded with Ju and Qu (Figure 4).

Figure 4 shows the Raman spectra for GO support loaded with the bioactive agents Ju and Qu. Raman spectroscopy can highlight structural changes and can offer information related to the number of layers presented in the GO materials. The disorder in GO can be determined by measuring the intensity ratio corresponding to D and G bands [48,49]. The Raman spectrum for GO presented in a previous work [20] is similar to the spectra presented in other literature reports [50,51,52]. The peaks around 1340 and 1610 cm^−1^ are representative for D and G bands in GO samples. After addition of the two biological active agents onto the GO nanocarrier, structural changes occur: the peak intensity of the D and G bands (corresponding to sp_3_ and sp_2_ carbon forms) is decreasing due to the enhancement of active compounds’ concentration, confirming their adsorption on the surface of GO. The slight increase of the I_D_/I_G_ ratio of GO-based nanomaterials (between 0.97 and 0.99, increasing in the order GO/Ju 5% < GO/Qu 2.5% < GO/Ju 10% < GO/Qu 5% (*w*/*v*)) when compared to GO (0.96) confirmed that the active compounds can repair the structure defects of graphene oxide during functionalization [36]. The peak intensity of the D band for the two DDS is increased, which confirmed the addition of the two polyphenols onto the surface of GO [19,48].

The 2D band can be used to determine the number of layers in GO nanomaterials. If the 2D band appears as a single peak, it is considered to indicate single-layer GO. An increase in the number of layers increases the number of 2D bands and reduces the intensities of the 2D peaks [35]. The 2D band occurs in the range of 2550 and 3390 cm^−1^. The ratio I_2D_/I_G_ for all samples is between 0.70 for GOJu 5% (*w*/*v*) and 0.84 for GOQu 5% (*w*/*v*). These results indicate that GO materials have a multilayer form [53,54].

### 3.4. MALDI-HRMS Analysis

**Figure 5:**
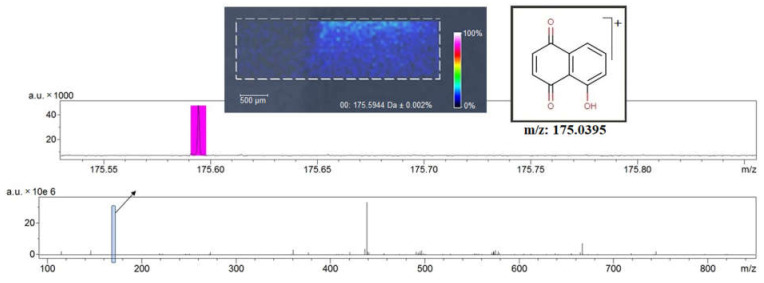

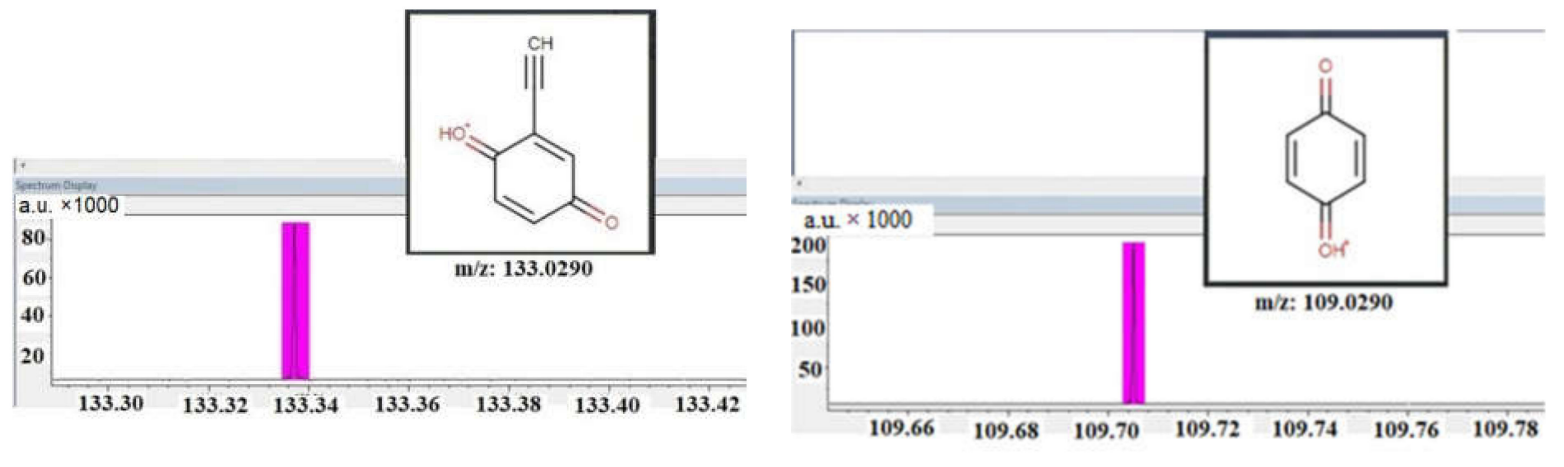
MALDI analysis of GOJu nanofilm.

**Figure 6:**
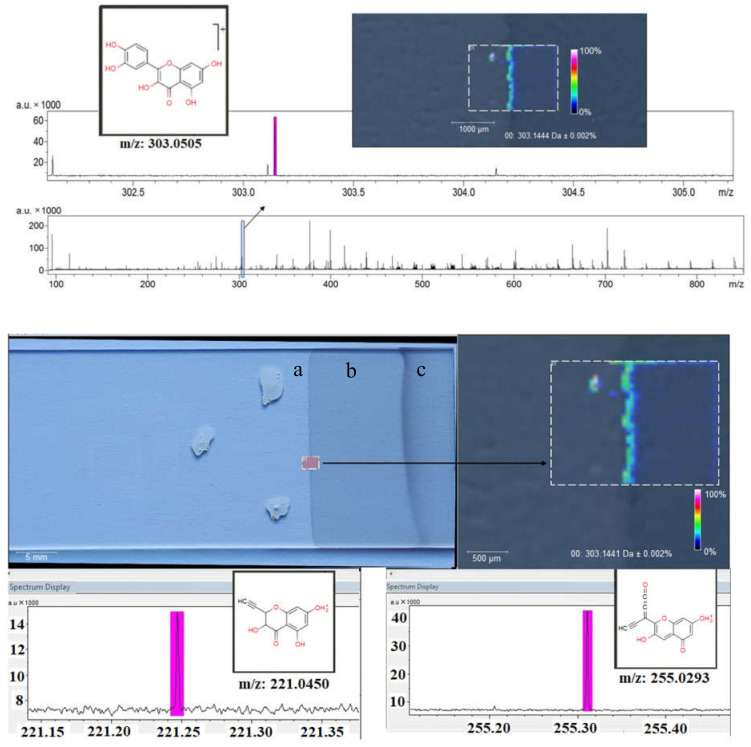
MALDI analysis of GOQu nanofilm showing the starting (**a**), middle (**b**), and end (**c**) region of the nanofilm.

The positive ionization using MALDI indicated the compounds’ mass with high accuracy, thus confirming the identity of natural substances Qu and Ju. No further matrix spraying steps were required because of the specific properties of GO, known as a versatile matrix for the MALDI analysis [55]. The MALDI images prove a good nanofilm homogeneity and an efficient transfer of the bioactive compound onto the nanofilm surface by the dip-coating process, as well as an efficient adsorption of Qu and Ju on the GO matrix. This is illustrated in Figure 5 and Figure 6, showing the fragmentation spectrum of Ju and Qu (m/z 175 and 303, respectively) [56]. All obtained nanofilms show, as expected, small inhomogeneous regions at the starting point, because of the inherent drawing speed variation in the transitory regime (Figure 5).

The dip-coating drawing process yields good nanofilm homogeneity, except for the starting line and the end region (Figure 6a,c). The latter is related to the surface solvent accumulation at the plate extraction from the liquid dispersion (due to the interfacial forces from liquid to glass). This region was excluded from the MALDI analysis. Because of the use of continuous support strips in technical dip-coating applications, the transitory start-stop regions are easy to remove by cutting. Therefore, our main interest was focused only on the middle (Figure 6b) region of the obtained nanofilms.

### 3.5. Release Behavior of Active Compounds

**Figure 7:**
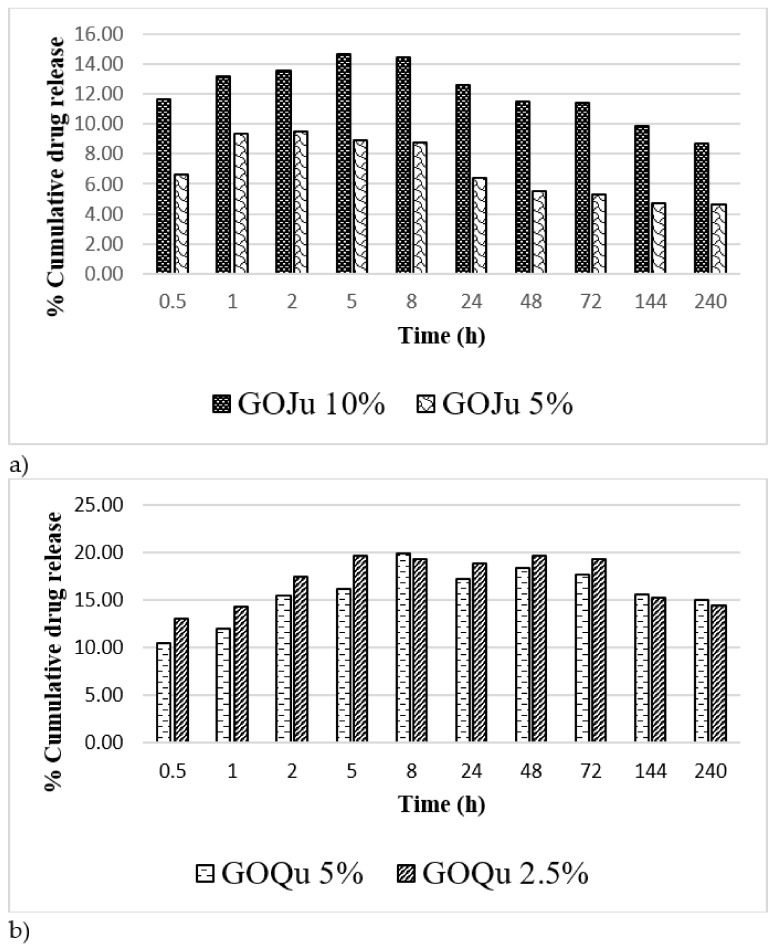
The release profile for (**a**) GOJu 10% (*w*/*v*) and GOJu 5% (*w*/*v*), and (**b**) GOQu 5% (*w*/*v*) and GOQu 2.5% (*w*/*v*).

The release profile of Qu and Ju from the GO-based materials was tested in SBF solution. Figure 7a,b present the drug release profiles at various time intervals of Qu and Ju from the GO-based DDS, for a period of up to 240 h. Ju was released quite fast in the first hours (Figure 7a), reaching a maximum release rate in the first 5 h of 14.6% for GOJu 10% (*w*/*v*) and of 9.5% in the first 2 h for GOJu 5% (*w*/*v*). The fast initial release of the substance can be a result of fast diffusion of the loosely bound drug molecules at the top part of the GO. After the fast release, GOJu 10% (*w*/*v*) nanocarrier exhibited a sustained/gradual and slow release, compared to the initial burst release of Ju from GOJu 5% (*w*/*v*). This could have been due to the small amount of Ju entrapped in cavities of the GO support, which may have reduced the diffusion of Ju. In vitro release studies showed a higher release ratio for GOJu 10% (*w*/*v*) compared to GOJu 5% (*w*/*v*), demonstrating that the release of the drug increases with the increase in the amount of Ju. After 8 h, a slight resorption of the drug was observed that might be associated with the ion exchange occurring in the buffer media between the anions in the drug and the GO nanocarrier. On the other hand, the re-absorption of the drug could be related to hydrophobic attractions and hydrogen bonding between GO and drug [57].

The release profile of Qu from GOQu 2.5% (*w*/*v*) and GOQu 5% (*w*/*v*) was similar under the same conditions, the maximum release ratio reaching 19.9% at 8 h for GOQu 5% (*w*/*v*) and 19.6% at 5 h for GOQu 2.5% (*w*/*v*). After 8 h, the release rate achieved an equilibrium state [19,58]. This can be attributed to drug entrapment in cavities and chemical interactions between negative groups of GO and drug, which may have reduced the diffusion of Qu. These results confirmed that GO-based materials exhibited a good sustainable release profile, because of which they can be used as a drug delivery system. This can be adequate to achieve a desirable local concentration release of the drugs during the initial few hours [59,60].

### 3.6. Evaluation of Antimicrobial Activity

**Figure 8:**
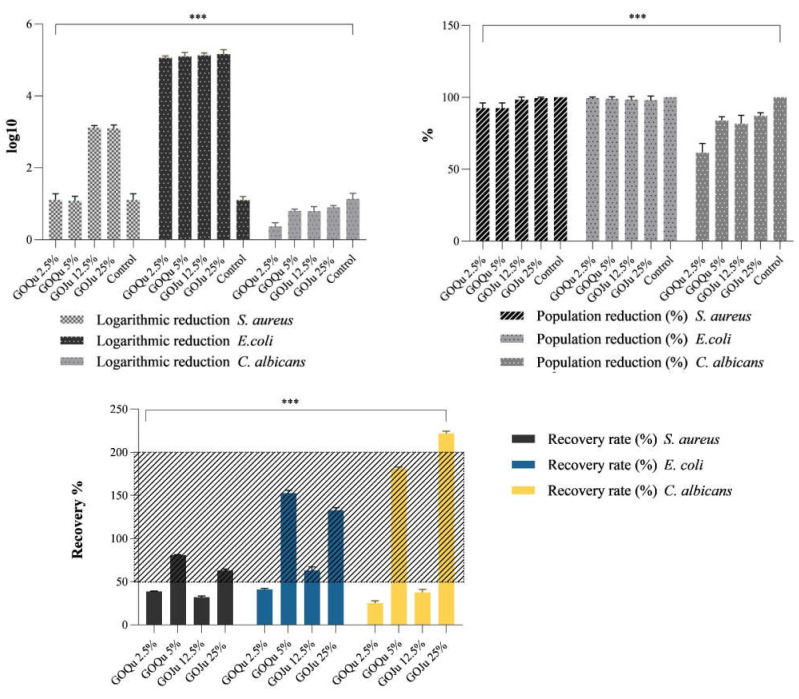
Logarithmic reduction, population reduction, and recovery rate of *S. aureus* ATCC, *E. coli* ATCC, and *C. albicans* ATCC strains by samples GOQu 5% (*w*/*v*), GOQu 2.5% (*w*/*v*), GOJu 10% (*w*/*v*), and GOJu 5% (*w*/*v*). Control is represented by microbial strains with no treatment; *p*-value < 0.002 (***).

Samples of Qu and Ju loaded on GO as support were evaluated in the presence of Gram-positive, Gram-negative, and microfungi reference strains. Logarithmic factor 1 was considered as a control and represents a 90% efficiency in bacterial reduction. The results on logarithmic reduction presented in Figure 8 show a 5.09 ± 0.06 log reduction for the *E. coli* strain, the samples being most effective on this Gram-negative strain. On *S. aureus,* the logarithmic reduction was 1.01 ± 0.1 for samples GOQu 2.5% (*w*/*v*) and GOQu 5% (*w*/*v*) and 3.04 ± 0.07 for the samples GOJu 5% (*w*/*v*) and GOJu 10% (*w*/*v*); they were not concentration sensitive in this range of concentrations. Obtained results indicate excellent antimicrobial efficiency for the samples loaded with Ju, especially on *E. Coli*. Results obtained for the *C. albicans* strain indicate a reduction ranging from 0.36 ± 0.08 to 0.94 ± 0.04 log, which is relatively modest compared to the values obtained on Gram strains.

In correlation with the logarithmic reduction, the population reduction (%) results for all strains tested are presented in Figure 8. A 100% reduction was considered as control. On *S. aureus* and *E. coli* strains, all samples manifested excellent antimicrobial activity, with reduction rates ranging from 92.49 ± 2.49% to 99.49 ± 0.5%. As for the *C. albicans* strain, reduction percentages were 57.1 ± 2.4% for sample GOQu 2.5% (*w*/*v*), 85.7 ± 1.8% for GOQu 5% (*w*/*v*), 42.8 ± 2.7% for GOJu 5% (*w*/*v*), and 63.7 ± 1.2% for sample GOJu 10% (*w*/*v*).

As the European Pharmacopoeia recommends for antimicrobial substances, the recovery rate must not differ by more than two factors (50–200% recovery). Samples GOQu 5% (*w*/*v*) and GOJu 10% (*w*/*v*) fall within the indicated range for the *S. aureus* strain. In addition, sample GOJu 5% (*w*/*v*) falls within the recommended range for *E. coli,* but not for *C. albicans* and *S. aureus* strains. Therefore, for samples GOQu 5% (*w*/*v*) and GOJu 10% (*w*/*v*), the antimicrobial efficiency is demonstrated and correlated as a trend with the logarithmic and percentage reduction results.

**Figure 9:**
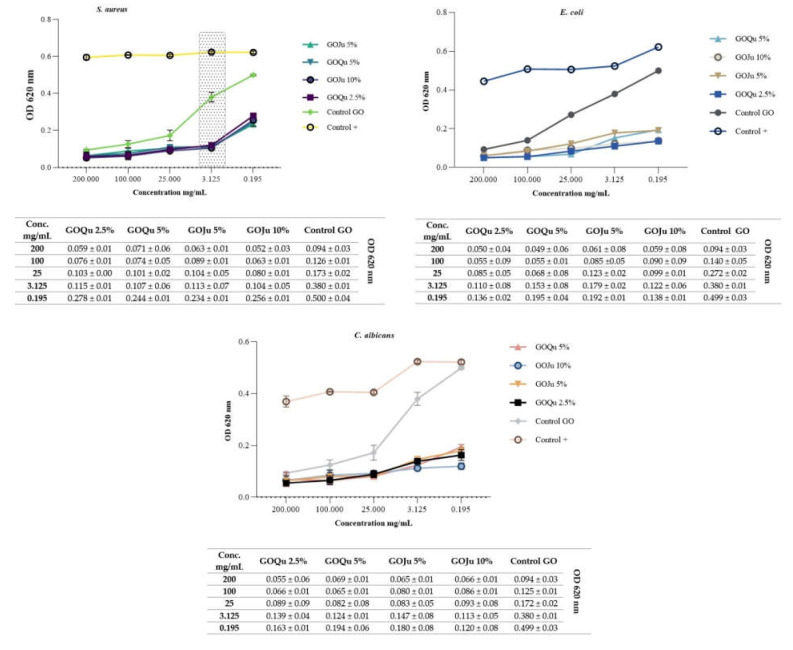
Trendline of antimicrobial susceptibility concentrations of samples GOQu 5% (*w*/*v*), GOQu 2.5% (*w*/*v*), GOJu 10% (*w*/*v*), and GOJu 5% (*w*/*v*). Control + represents the microbial strains with no treatment.

Antimicrobial susceptibility was evaluated by the broth microdilution method, against *S. aureus*, *E. coli*, and *C. albicans* strains, all being considered representative microorganisms for their group and significant pathogens associated with various post-operatory infections. For this assay, solutions with the following concentrations were tested: 200 mg/mL, 100 mg/mL, 25 mg/mL, 3.125 mg/mL, and 0.195 mg/mL. In order to determine the most effective antimicrobial concentrations, we considered an optimal ratio between concentrations with the lowest cytotoxicity (C) and with the highest antimicrobial effect (A). The results obtained and presented in Figure 9 indicate that for *S. aureus*, all samples tested present the most suitable correlation of C and A at 3.125 mg/mL. The GO control activity is improved by functionalization with Qu and Ju. For *E. coli*, the best C/A ratio is obtained at 0.195 mg/mL for samples GOQu 2,5%, GOQu 5%, and GOJu10%. For sample GOJu 5%, the 3.125 mg/mL concentration was optimal. For *C. albicans* strain, the values with good C/A ratio were obtained at 0.195 mg/mL for samples GOQu 2.5% and GOJu 10%, and at 3.125% for samples GO Qu 5% and GOJu 5% [61]. The GO control presented improved antimicrobial efficacy when tested on *E.coli* and *C. albicans* strains.

When evaluated with the biocompatibility assay taken into consideration, the results obtained indicate that the 0.195 mg/mL concentration is the most suitable for optimal therapeutic effect vs. safety of use.

The obtained results correlate properly and indicate good antimicrobial efficacy on *S. aureus* and *E. coli* strains, especially for samples GOQu 5% (*w*/*v*) and GOJu 10% (*w*/*v*). As for the *Candida* strain, the obtained effect indicates fungistatic action, with samples reducing the microfungi populations from 42.8 ± 2.7% to 85.7 ± 1.8%.

### 3.7. Biocompatibility Tests

The cellular viability and proliferation of both normal (L929, fibroblasts) and tumor (BT474, breast cancer) cells, after 48 h of treatment, were assessed for GO. The investigations were performed in comparison with the free active substance in equivalent concentrations to conjugated GO. Similarly, the non-conjugated GO cytotoxicity (in equivalent concentrations to conjugated GO) was investigated for the two cell lines, at 48 h.

**Figure 10:**
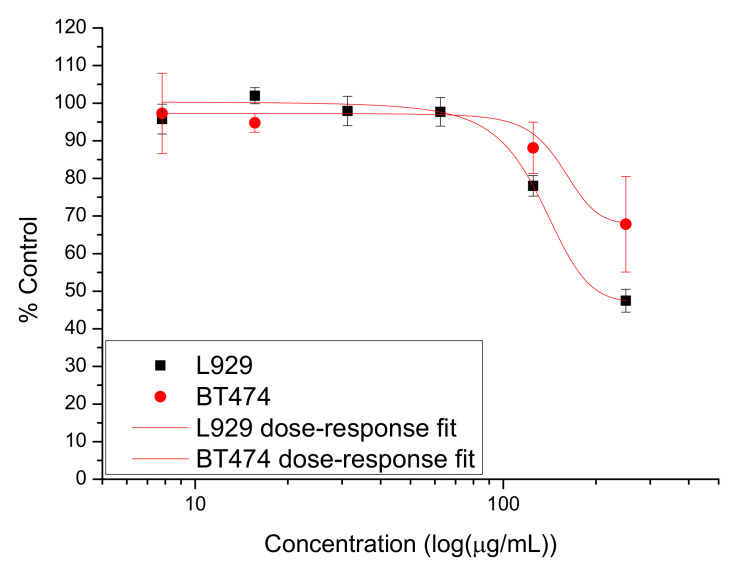
Cytotoxicity of GO on L929 (black square) and BT474 (red dot) cells: proliferation of cells incubated with GO over 48 h. Data are calculated as percentage of untreated control and are shown as mean ± SD and represented as dependent on the drug concentration decimal logarithm. The red curves have been obtained using the dose–response fit in OriginPro 8.6.

Drug-free GO showed a biocompatible behavior for both L929 and BT474 cells at lower concentrations (Figure 10), as the viability was above 70% compared to control cells. The highest concentrations employed (125 and 250 μg/mL, respectively) showed a slight cytotoxic behavior (the viability was below 70% compared to controls). The effect was not statistically significant (*p* > 0.05) in the case of BT474 cells. For fibroblast cells, a decrease of the viability by 52.52 ± 3.05% (*p* = 0.002) compared to control cells was determined for the highest concentration employed in the study.

**Figure 11:**
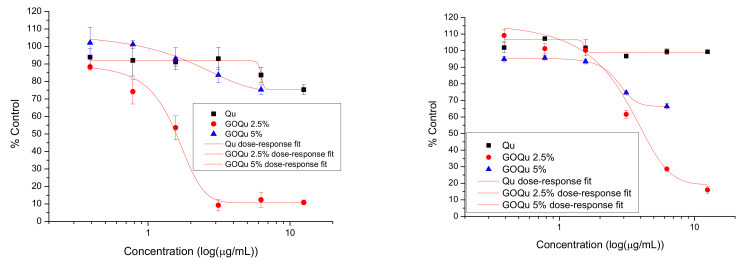
Cytotoxicity of GOQu on L929 (**left**) and BT474 (**right**) cells: proliferation of cells incubated with Qu (black sqare), GOQu 5% (*w*/*v*) (blue triangle), and GOQu 2.5% (*w*/*v*) (red dot), in equivalent concentrations, over 48 h. Data are calculated as percentage of untreated control and are shown as mean ± SD and represented as dependent on the drug concentration decimal logarithm. The red curves have been obtained using the dose–response fit in OriginPro 8.6.

Free Qu showed a biocompatible behavior for both L929 and BT474 cells at all concentrations (Figure 11). GOQu 5% (*w*/*v*) showed a biocompatible behavior for L929 cells at all investigated concentrations, but in the case of BT474, a slight cytotoxic effect at the highest concentrations was determined (*p* < 0.05). On the other hand, GOQu 2.5% (*w*/*v*) showed a cytotoxic effect for both L929 and BT474 cells (*p* < 0.05). The effect was accentuated in the case of normal fibroblast cells, compared to breast cancer (*p* < 0.01), because the dose–response curve had a drop from the low concentrations. The EC50 in the case of GOQu 2.5% (*w*/*v*) was 70.07 ± 36.76 (L929) and 99.29 ± 0.07 (BT474), while in the case of GOQu 5% (*w*/*v*), it was 35.04 ± 0.53 (L929) and 5.98 ± 0.75 (BT474). The therapeutic index for GOQu 2.5% (*w*/*v*) was 1.417 ± 0.002, and for GOQu 5%, 0.17 ± 1.41 (*w*/*v*).

**Figure 12:**
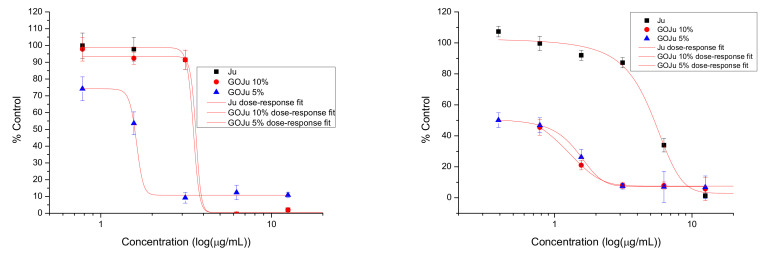
Cytotoxicity of GOJu on L929 (**left**) and BT474 (**right**) cells: proliferation of cells incubated with Ju (black square), GOJu 10% (*w*/*v*) (red dot), and GOJu 5% (*w*/*v*) (blue triangle), in equivalent concentrations, over 48 h. Data are calculated as percentage of untreated control and are shown as mean ± SD and represented as dependent on the drug concentration decimal logarithm. The red curves have been obtained using the dose–response fit in OriginPro 8.6.

Free Ju showed a similar effect for both fibroblast and breast cancer cells at all concentrations (EC50 for L929: 3.55 ± 0.06; for BT474: 4.77 ± 0.08)(Figure 12). In the case of L929, GOJu 10% (*w*/*v*) showed a similar effect to free J (NS), while GOJu 5% (*w*/*v*) showed an increased cytotoxicity compared to both Ju (*p* < 0.05) and GOJu 10% (*w*/*v*) (*p* < 0.01). The EC50 for GOJu 10% (*w*/*v*) was 35.04 ± 0.44, for GOJu 5% (*w*/*v*) 25.3 ± 0.28, and for Ju 3.55 ± 0.06. In the case of BT474, both GOJu 10% (*w*/*v*) (*p* < = 0.001 except the highest concentrations) and GOJu 5% (*w*/*v*) (*p* < 0.01) showed an increased cytotoxicity at all concentrations compared to free Ju. The EC50 was 5.98 ± 0.75 for GOJu 10% (*w*/*v*), 6.75 ± 0.86 for GOJu 5% (*w*/*v*), and 4.77 ± 0.08 for Ju. The therapeutic index was 1.34 ± 1.33 for Ju, 0.17 ± 1.7 for GOJu 10% (*w*/*v*), and 0.26 ± 3.07 for GOJu 5%.

For *S. aureus* strain, the EC50 for GOQu 2.5% was 65.75 ± 0.6; for GOQu 5%, it was 24.64 ± 0.37; for GOJu 5%, it was 18.59 ± 0.4; and for GOJu 10%, it was 25.44 ± 1.01. For *E.coli* strain, the EC50 for GOQu 2.5% was 70.62 ± 0.15; for GOQu 5%, it was 1.80 ± 1.15; for GOJu 5%, it was 18.59 ± 0.97; and for GOJu 10%, it was 25.44 ± 0.31. For *C. albicans* strain, the EC50 for GOQu 2.5% was 95.79 ± 1.4; for GOQu 5%, it was 45.18 ± 0.39; for GOJu 5%, it was 43.98 ± 0.51; and for GOJu 10%, it was 45.20 ± 1.94.

The therapeutic index (used as a quantitative measurement of the relative safety for developed products), assessed by comparing the EC50 for the antimicrobial susceptibility with the EC50 for the biocompatibility, is summarized in Table 1.

## 4. Conclusions

The scope of this paper was to obtain a GO-based DDS by loading Ju and Qu as bioactive agents, and the antimicrobial and biocompatibility was evaluated. The obtained nanocomposites demonstrated excellent antimicrobial efficacy on both *S. aureus* and *E. coli* strains, with reduction rates above 90%, but a follow-up study with determination of minimal inhibitory concentrations will be performed. The release behavior results demonstrated potential for the designed platforms to be used as a DDS, having the capacity to ensure sustained release. GO-based DDS showed a biocompatible behavior for both L929 and BT474 cells at lower concentrations. Moreover, GOJu and GOQu with higher concentrations of active substances showed biocompatible behavior for L929 and BT474 cells, as compared to the nanoformulation with lower concentrations of substances. The MALDI analysis confirmed the identity of Qu and Ju and demonstrated an efficient transfer of the bioactive compound on the nanofilm surface. In addition, specific properties of GO made it a versatile matrix for the MALDI analysis. The results of this study indicate that GO-based materials are suitable for delivering the hydrophobic biological active agents Qu and Ju, proving biocompatibility and antibacterial activity.

## Figures and Tables

**Table 1:** Therapeutic index of the novel drug delivery system.

Samples		GOQu 2.5%	GOQu 5%	GOJu 5%	GOJu 10%
Microbial strains	*S. aureus*	0.93 ± 0.84	0.70 ± 0.50	0.73 ± 0.48	0.72 ± 0.60
	*E. coli*	1.01 ± 0.45	0.05 ± 0.02	1.08 ± 0.31	0.56 ± 0.22
	*C. albicans*	1.36 ± 0.11	1.28 ± 1.02	1.74 ± 0.02	1.29 ± 0.17

## Data Availability

The data presented in this study are available on request from the corresponding author.

## References

[1] Jampilek J., Kralova K. (2021). Advances in Drug Delivery Nanosystems Using Graphene-Based Materials and Carbon Nanotubes. Materials.

[2] Seema D.M.J., Saifullah B., Selvanayagam M., Gothai S., Hussein M.Z., Subbiah S.K., Esa N.M., Arulselvan P. (2018). Designing of the Anticancer Nanocomposite with Sustained Release Properties by Using Graphene Oxide Nanocarrier with Phenethyl Isothiocyanate as Anticancer Agent. Pharmaceutics.

[3] Valentini F., Calcaterra A., Ruggiero V., Pichichero E., Martino A., Iosi F., Bertuccini L., Antonaroli S., Mardente S., Zicari A. (2019). Functionalized Graphene Derivatives: Antibacterial Properties and Cytotoxicity. J. Nanomater..

[4] Rahman H., Othman H.H., Hammadi N.I., Yeap S.K., Amin K.M., Samad N.A., Alitheen N.B. (2020). Novel Drug Delivery Systems for Loading of Natural Plant Extracts and Their Biomedical Applications. Int. J. Nanomed..

[5] Popa B.V., Bratu A.M., Minoiu C.A., Turculet C.S., Ene D., Constantinescu G., Ilie M., Popescu M., Badila E. (2015). The Role of Lipiodol in the Treatment of Hepatocellular Carcinoma (HCC) through Transarterial Chemoembolization (TACE). Rev. De Chim. Buchar..

[6] Vogus D.R., Krishnan V., Mitragotri S. (2017). A review on engineering polymer drug conjugates to improve combination chemotherapy. Curr. Opin. Colloid Interface Sci..

[7] Liang W., Huang Y., Lu D., Ma X., Gong T., Cui X., Yu B., Yang C., Dong C., Shuang S. (2019). β-Cyclodextrin–Hyaluronic Acid Polymer Functionalized Magnetic Graphene Oxide Nanocomposites for Targeted Photo-Chemotherapy of Tumor Cells. Polymers.

[8] Pooresmaeil M., Namazi H. (2018). beta-Cyclodextrin grafted magnetic graphene oxide applicable as cancer drug delivery agent: Synthesis and characterization. Mater. Chem. Phys..

[9] Pooresmaeil M., Namazi H. (2018). Surface modification of graphene oxide with stimuli-responsive polymer brush containing beta-cyclodextrin as a pendant group: Preparation, characterization, and evaluation as controlled drug delivery agent. Colloids Surf. B: Biointerfaces.

[10] Croitoru A.M., Karacelebi Y., Saatcioglu E., Altan E., Ulag S., Aydogan H.K., Sahin A., Motelica L., Oprea O., Tihauan B.M. (2021). Electrically Triggered Drug Delivery from Novel Electrospun Poly(Lactic Acid)/Graphene Oxide/Quercetin Fibrous Scaffolds for Wound Dressing Applications. Pharmaceutics.

[11] Pan Y., Sahoo N.G., Li L. (2012). The application of graphene oxide in drug delivery. Expert Opin. Drug Deliv..

[12] Tiwari H., Karki N., Pal M., Basak S., Verma R.K., Bal R., Kandpal N.D., Bisht G., Sahoo N.G. (2019). Functionalized graphene oxide as a nanocarrier for dual drug delivery applications: The synergistic effect of quercetin and gefitinib against ovarian cancer cells. Colloids Surfaces B: Biointerfaces.

[13] Pandey S.K., Patel D.K., Thakur R., Mishra D.P., Maiti P., Haldar C. (2015). Anti-cancer evaluation of quercetin embedded PLA nanoparticles synthesized by emulsified nanoprecipitation. Int. J. Biol. Macromol..

[14] Wang P.W., Henning S.M., Magyar C.E., Elshimali Y., Heber D., Vadgama J.V. (2016). Green tea and quercetin sensitize PC-3 xenograft prostate tumors to docetaxel chemotherapy. J. Exp. Clin. Cancer Res..

[15] Sun D.D., Li N.A., Zhang W.W., Yang E.D., Mou Z.P., Zhao Z.W., Liu H.P., Wang W.Y. (2015). Quercetin-loaded PLGA nanoparticles: A highly effective antibacterial agent in vitro and anti-infection application in vivo. J. Nanopart. Res..

[16] Arasoglu T., Derman S., Mansuroglu B., Yelkenci G., Kocyigit B., Gumus B., Acar T., Kocacaliskan I. (2017). Synthesis, characterization and antibacterial activity of juglone encapsulated PLGA nanoparticles. J. Appl. Microbiol..

[17] Ji Y.-B., Qu Z.-Y., Zou X. (2011). Juglone-induced apoptosis in human gastric cancer SGC-7901 cells via the mitochondrial pathway. Exp. Toxicol. Pathol..

[18] Sarkar A., Ghosh S., Chowdhury S., Pandey B., Sil P.C. (2016). Targeted delivery of quercetin loaded mesoporous silica nanoparticles to the breast cancer cells. Biophys. Acta (BBA) Gen. Subj..

[19] Islami M., Zarrabi A., Tada S., Kawamoto M., Isoshima T., Ito Y. (2018). Controlled quercetin release from high-capacity-loading hyperbranched polyglycerol-functionalized graphene oxide. Int. J. Nanomed..

[20] Zheng Y., He L., Asiamah T.K., Otto M. (2018). Colonization of Medical Devices by Staphylococci. Environ. Microbiol..

[21] Spampinato C., Leonardi D. (2013). CandidaInfections, Causes, Targets, and Resistance Mechanisms: Traditional and Alternative Antifungal Agents. BioMed Res. Int..

[22] Payne V.K., Florence Cécile T.T., Cedric Y., Christelle Nadia N.A., José O. (2020). Risk Factors Associated with Prevalence of Candida albicans, Gardnerella vaginalis, and Trichomonas vaginalis among Women at the District Hospital of Dschang, West Region, Cameroon. Int. J. Microbiol..

[23] Anh D.N., Hung D.N., Tien T.V., Dinh V.N., Son V.T., Luong N.V., Quynh N.T.N., Van Tuan N., Tuan L.Q., Bac N.D. (2021). Prevalence, species distribution and antifungal susceptibility of Candida albicans causing vaginal discharge among symptomatic non-pregnant women of reproductive age at a tertiary care hospital, Vietnam. BMC Infect. Dis..

[24] Mofolorunsho K.C., Ocheni H.O., Aminu R.F., Omatola C.A., Olowonibi O.O. (2021). Prevalence and antimicrobial susceptibility of extended-spectrum beta lactamases-producing Escherichia coli and Klebsiella pneumoniae isolated in selected hospitals of Anyigba, Nigeria. Afr. Health Sci..

[25] Wu M., Tong X., Liu S., Wang D., Wang L., Fan H. (2019). Prevalence of methicillin-resistant Staphylococcus aureus in healthy Chinese population: A system review and meta-analysis. PLoS ONE.

[26] Ayeni F.A. (2018). Prevalence, Diagnosis and Local Susceptibility of Staphylococci Infections. Staphylococcus Aureus.

[27] Rodríguez I., Figueiredo A.S., Sousa M., Aracil-Gisbert S., Fernández-De-Bobadilla M.D., Lanza V.F., Rodríguez C., Zamora J., Loza E., Mingo P. (2021). A 21-Year Survey of Escherichia coli from Bloodstream Infections (BSI) in a Tertiary Hospital Reveals How Community-Hospital Dynamics of B2 Phylogroup Clones Influence Local BSI Rates. mSphere.

[28] Khatoon Z., McTiernan C.D., Suuronen E.J., Mah T.-F., Alarcon E.I. (2018). Bacterial biofilm formation on implantable devices and approaches to its treatment and prevention. Heliyon.

[29] Almeida G.C.M., dos Santos M.M., Lima N.G.M., Cidral T.A., Melo M.C.N., Lima K.C. (2014). Prevalence and factors associated with wound colonization by Staphylococcus spp. and Staphylococcus aureus in hospitalized patients in inland northeastern Brazil: A cross-sectional study. BMC Infect. Dis..

[30] Croitoru A., Oprea O., Nicoara A., Trusca R., Radu M., Neacsu I., Ficai D., Ficai A., Andronescu E. (2019). Multifunctional Platforms Based on Graphene Oxide and Natural Products. Medicina.

[31] Kokubo T., Takadama H. (2006). How useful is SBF in predicting in vivo bone bioactivity?. Biomaterials.

[32] CLSI (2018). M07—Methods for Dilution Antimicrobial Susceptibility Tests for Bacteria That Grow Aerobically.

[33] Wang K., Ruan J., Song H., Zhang J., Wo Y., Guo S., Cui D. (2011). Biocompatibility of Graphene Oxide, Nanoscale research letters. Nanoscale Res. Lett..

[34] Han W., Niu W.Y., Sun B., Shi G.C., Cui X.Q. (2016). Biofabrication of polyphenols stabilized reduced graphene oxide and its anti-tuberculosis activity, Journal of photochemistry and photobiology. J. Photochem. Photobiol. B: Biol..

[35] Aliyev E., Filiz V., Khan M.M., Lee Y.J., Abetz C., Abetz V. (2019). Structural Characterization of Graphene Oxide: Surface Functional Groups and Fractionated Oxidative Debris. Nanomaterials.

[36] Pavia D.L., Lampan G.M., George K.R.Z. (2001). Introduction to Spectroscopy.

[37] Al-Attar M.S. (2017). Green Chemistry Reactions in Duhok City: Part I. Rose Bengal Solar Photosensitized Synthesis of Juglone. Zanco J. Pure Appl. Sci..

[38] Bennet D., Marimuthu M., Kim S., An J. (2012). Dual drug-loaded nanoparticles on self-integrated scaffold for controlled delivery. Int. J. Nanomed..

[39] Porto I.C.C.M., Nascimento T.G., Oliveira J.M.S., Freitas P.H., Haimeur A., Franca R. (2018). Use of polyphenols as a strategy to prevent bond degradation in the dentin-resin interface. Eur. J. Oral Sci..

[40] Catauro M., Papale F., Bollino F., Piccolella S., Marciano S., Nocera P., Pacifico S. (2015). Silica/quercetin sol-gel hybrids as antioxidant dental implant materials. Sci. Technol. Adv. Mat..

[41] Saqezi A.S., Kermanian M., Ramazani A., Sadighian S. (2022). Synthesis of Graphene Oxide/Iron Oxide/Au Nanocomposite for Quercetin Delivery. J. Inorg. Organomet. Polym. Mater..

[42] Singh D., Rawat M.S., Semalty A., Semalty M. (2012). Quercetin-phospholipid complex: An amorphous pharmaceutical system in herbal drug delivery. Curr. Drug Discov. Technol..

[43] Song J.G., Wang X.Z., Chang C.T. (2014). Preparation and Characterization of Graphene Oxide. J. Nanomater..

[44] Zaaba N.I., Foo K.L., Hashim U., Tan S.J., Liu W.W., Voon C.H. (2017). Synthesis of Graphene Oxide using Modified Hummers Method: Solvent Influence. Adv. Mater. Processing Technol. Conf..

[45] Zhang Q., Huang X., Pu Y.Q., Yi Y.X., Zhang T., Wang B. (2018). pH-sensitive and biocompatible quercetin-loaded GO-PEA-HA carrier improved antitumour efficiency and specificity. Artif. Cell Nanomed. B.

[46] Yuan Y.G., Wang Y.H., Xing H.H., Gurunathan S. (2017). Quercetin-mediated synthesis of graphene oxide-silver nanoparticle nanocomposites: A suitable alternative nanotherapy for neuroblastoma. Int. J. Nanomed..

[47] Long J.P., Li S.X., Liang J.M., Wang Z.G., Liang B. (2019). Preparation and characterization of graphene oxide and it application as a reinforcement in polypropylene composites. Polym. Compos..

[48] Jiang Z.Y., Jin H.L., Sun S., Chen C.Q., Zhang J., Guo Z.F., Liu X.Y. (2018). Effects of gallic acid biofabricated rGO nanosheets combined with radiofrequency radiation for the treatment of renal cell carcinoma. Mater. Sci. Eng. C.

[49] Gurunathan S., Han J.W., Dayem A.A., Eppakayala V., Kim J.H. (2012). Oxidative stress-mediated antibacterial activity of graphene oxide and reduced graphene oxide in Pseudomonas aeruginosa. Int. J. Nanomed..

[50] Johra F.T., Lee J.W., Jung W.G. (2014). Facile and safe graphene preparation on solution based platform. J. Ind. Eng. Chem..

[51] Eremina E.A., Kaplin A.V., Eliseev A.A., Sidorov A.V., Radzhabzoda S.S., Grigor’eva A.V., Gudilin E.A. (2018). Multifunctional Composites Based on Graphite Oxide, Doxorubicin, and Magnetic Nanoparticles for Targeted Drug Delivery. Nanotechnol. Russ..

[52] Kordi F., Zak A.K., Darroudi M., Saedabadi M.H. (2019). Synthesis and characterizations of Ag-decorated graphene oxide nanosheets and their cytotoxicity studies. Chem. Pap..

[53] Muzyka R., Drewniak S., Pustelny T., Chrubasik M., Gryglewicz G. (2018). Characterization of Graphite Oxide and Reduced Graphene Oxide Obtained from Different Graphite Precursors and Oxidized by Different Methods Using Raman Spectroscopy. Materials.

[54] Minitha C.R., Rajendrakumar R. (2013). Synthesis and Characterization of Reduced Graphene Oxide. Adv. Mater. Res..

[55] Wang Z.J., Cai Y., Wang Y., Zhou X.W., Zhang Y., Lu H.J. (2017). Improved MALDI imaging MS analysis of phospholipids using graphene oxide as new matrix. Sci. Rep..

[56] Scigelova M., Hornshaw M., Giannakopulos A., Makarov A. (2011). Fourier Transform Mass Spectrometry. Mol. Cell Proteom..

[57] Wang H., Wang C.P., Zou Y., Hu J.J., Li Y.W., Cheng Y.Y. (2020). Natural polyphenols in drug delivery systems: Current status and future challenges. Giant.

[58] Luzi F., Pannucci E., Santi L., Kenny J.M., Torre L., Bernini R., Puglia D. (2019). Gallic Acid and Quercetin as Intelligent and Active Ingredients in Poly(vinyl alcohol) Films for Food Packaging. Polymers.

[59] Mohan L., Anandan C., Rajendran N. (2016). Drug release characteristics of quercetin-loaded TiO2 nanotubes coated with chitosan. Int. J. Biol. Macromol..

[60] Matiyani M., Rana A., Pal M., Rana S., Melkani A.B., Sahoo N.G. (2022). Polymer grafted magnetic graphene oxide as a potential nanocarrier for pH-responsive delivery of sparingly soluble quercetin against breast cancer cells. Rsc. Adv..

[61] Andrews J.M. (2001). Determination of minimum inhibitory concentrations. J. Antimicrob. Chemoth..

